# Cellular Adhesion Gene *SELP* Is Associated with Rheumatoid Arthritis and Displays Differential Allelic Expression

**DOI:** 10.1371/journal.pone.0103872

**Published:** 2014-08-22

**Authors:** Jana Burkhardt, Mechthild Blume, Elisabeth Petit-Teixeira, Vitor Hugo Teixeira, Anke Steiner, Elfi Quente, Grit Wolfram, Markus Scholz, Céline Pierlot, Paola Migliorini, Stefano Bombardieri, Alejandro Balsa, René Westhovens, Pilar Barrera, Timothy R. D. J. Radstake, Helena Alves, Thomas Bardin, Bernard Prum, Frank Emmrich, François Cornelis, Peter Ahnert, Holger Kirsten

**Affiliations:** 1 Translational Centre for Regenerative Medicine (TRM), Universität Leipzig, Leipzig, Germany; 2 University Leipzig, Institute for Clinical Immunology and Tranfusion Medicine (IKIT), Universität Leipzig, Leipzig, Germany; 3 Genhotel-EA 3886, Université d'Evry-Val d'Essonne, Evry, France; 4 Centre for Respiratory Research, University College London, London, United Kingdom; 5 Fraunhofer Institute for Celltherapy and Immunology (IZI), Leipzig, Germany; 6 LIFE – Leipzig Research Center for Civilization Diseases, Universität Leipzig, Leipzig, Germany; 7 Pisa University, Pisa, Italy; 8 La Paz Hospital, Madrid, Spain; 9 Rheumatology KU Leuven, Leuven, Belgium; 10 Nijmegen University, Nijmegen, the Netherlands; 11 University Medical Center Utrecht, Utrecht, the Netherlands; 12 Porto San Joao Hospital, Porto, Portugal; 13 Fédération de Rhumatologie, Lariboisière Hospital, Paris, France; 14 Laboratoire Statistique & Génome, Université d'Evry-Val d'Essonne, Evry, France; 15 GenHotel-Auvergne, Université d'Auvergne, Clermont-Ferrand, France; 16 Institute for Medical Informatics, Statistics and Epidemiology (IMISE), Universität Leipzig, Leipzig, Germany; Keio University School of Medicine, Japan

## Abstract

In rheumatoid arthritis (RA), a key event is infiltration of inflammatory immune cells into the synovial lining, possibly aggravated by dysregulation of cellular adhesion molecules. Therefore, single nucleotide polymorphisms of 14 genes involved in cellular adhesion processes (*CAST, ITGA4, ITGB1, ITGB2, PECAM1, PTEN, PTPN11, PTPRC, PXN, SELE, SELP, SRC, TYK2, and VCAM1*) were analyzed for association with RA. Association analysis was performed consecutively in three European RA family sample groups (N_families_ = 407). Additionally, we investigated differential allelic expression, a possible functional consequence of genetic variants. *SELP* (selectin P, CD62P) SNP-allele rs6136-T was associated with risk for RA in two RA family sample groups as well as in global analysis of all three groups (p_total_ = 0.003). This allele was also expressed preferentially (p<10^−6^) with a two- fold average increase in regulated samples. Differential expression is supported by data from Genevar MuTHER (p_1_ = 0.004; p_2_ = 0.0177). Evidence for influence of rs6136 on transcription factor binding was also found *in silico* and in public datasets reporting *in vitro* data. In summary, we found SELP rs6136-T to be associated with RA and with increased expression of SELP mRNA. SELP is located on the surface of endothelial cells and crucial for recruitment, adhesion, and migration of inflammatory cells into the joint. Genetically determined increased SELP expression levels might thus be a novel additional risk factor for RA.

## Introduction

Rheumatoid Arthritis (RA) is a chronic inflammatory disease with features of an autoimmune disease [1]. There is ample evidence for genetic influences on RA and heritability is estimated to be about 60% [2]. It is estimated that risk alleles identified up to now explain about 17% of seropositive RA [3]. One hallmark of RA pathogenesis is infiltration of synovial fluid by autoreactive immune cells. These cells release inflammatory cytokines, immunoglobulins, and rheumatoid factor (RF). Macrophages ingest these RF immune complexes and release additional cytokines (e.g. IL1, IL6), leading to activation of the complement system and release of more inflammatory mediators and cartilage degrading enzymes. Macrophages as well as proliferating fibroblast-like cells induce typical joint swelling and pannus formation in the inflamed synovial membrane. This cycle of activation and infiltration of inflammatory cells, release of inflammatory mediators and action of aggressive cartilage degrading enzymes finally causes destruction of cartilage and joints. Hence, in the pathogenesis of RA, infiltration of inflammatory cells into the synovial lining plays an important role and may be modulated by dysregulation of cellular adhesion molecules [4], [5]. Adhesion molecules are expressed on the surface of cells and mediate adhesion of cells to other cells or to the extracellular matrix [6]. They can be divided into three superfamilies: selectins, integrins, and the Ig-superfamily. Adhesion molecules regulate leukocyte circulation, lymphoid cell homing to tissues and inflammatory sites, and transendothelial migration. They also participate in lymphocyte co-stimulation, cytotoxicity, lymphohemopoiesis, and B cell apoptosis. In RA, gene expression of integrins and their ligands were found to be up-regulated [7] and studies even suggest correlations with prognosis and disease activity for adhesion gene products such as selectin P which is encoded by the gene *SELP* [8], [9]. Therefore, genes involved in cellular adhesion processes are highly relevant candidate genes for studying genetic association with RA.

To identify genes involved in cellular adhesion relevant in RA, we performed a thorough literature and database analysis. We favored genes that are located in genomic regions identified in genome-wide linkage scans in RA families [10] and genes with polymorphisms known to be associated with autoimmune disorders. Table 1 displays relevant information on all 14 genes selected. In brief, some properties and functions of these genes are as follows: Integrin: Integrin ITGA4 is the alpha-4 subunit of integrin VLA-4, ITGB1 is the beta-1 subunit of the same molecule. VLA-4 is a cell surface receptor on activated lymphocytes and monocytes that binds the ligand VCAM1 on endothelial cells. It is particularly involved in firm adhesion prior to migration. ITGB2 is the beta-2 subunit of the integrin LFA-1, which is also expressed on lymphocytes, especially leukocytes. Ligands of LFA-1 are ICAM1, ICAM2, and VCAM1 on endothelial cells. TYK2 is a tyrosine kinase belonging to the group of Janus kinases. The molecule is part of the regulatory network of integrin mediated adhesion and migration by activating negative regulators upon signaling [11]. The *PXN* (*paxillin*) gene is located in an RA linkage region and part of the focal adhesion process. Its protein product, PXN is in direct contact with the focal adhesion kinase (FAK). PXN is part of growth factor and adhesion mediated cell signaling processes including migration, proliferation, and gene expression activation [12]. SRC is a kinase and part of the integrin signaling network. Its gene is highly regulated by multiple binding partners such as FAK. PTPRC is a binding partner and regulator of SRC. During integrin mediated adhesion, PTPRC is bound to the tyrosine kinase complex and inhibits SRC mainly via the kinase domain [13]. There are several association studies published for *PTPRC* with autoimmune diseases [14], [15]. *PTEN* and *PTPN11* both are tumor suppressor genes involved in migration processes. FAK is a substrate for PTEN and the FAK pathway influences organization of actin filaments during cellular migration [16]. PTPN11 is involved in Rho signaling, which is part of cellular adhesion processes. It is also a positive regulator of the SRC mediated integrin pathway [17]. Calpastatin (CAST) is an inhibitor of calpain, a protease involved in apoptosis, proliferation, and migration. This molecule is part of the regulatory network of integrin mediated cellular adhesion via RhoA and FAK signaling pathways as well as via direct binding to the beta subunit of integrins,. Additionally, calpain regulates integrin activation via talin, a prerequisite for firm adhesion. Inhibition of calpain has been shown to reduce cellular migration [18]. PECAM1 and VCAM1 belong to the Ig superfamily and are widely expressed on hematopoietic cells. The blocking of PECAM1 with specific antibodies reduces 90% of leukocyte migration. In combination with CD99 blocking, diapedesis is nearly completely inhibited [19]. SELE and SELP are adhesion proteins belonging to the selectin class and are expressed on the surface of activated endothelial cells. SELP (selectin P, CD62P) can be activated immediately due to its location in Weibel-Palade bodies, where it is stored along with von Willebrand factor. The function of selectins is the binding of blood-leukocytes on activated endothelial cells prior to migration in inflamed tissue and therefore it is relevant for autoimmune diseases and RA in particular. Circulating SELE is found to be increased in RA patients and correlates with disease progression [20]. SELP is up-regulated in atherosclerotic plaque and in patients with angina pectoris. The non-synonymous single nucleotide polymorphisms (SNP) rs6136 in SELP has previously been associated with myocardial infarction with the A allele representing a risk factor [21], [22]. RA patients may also carry a higher risk for myocardial infarction [23].

**Table 1:** Adhesion genes and SNPs selected for association study with RA.

Hugo gene ID	Gene name	RA linkage region*	Known associations	position	SNP IDs	Involvement in cellular adhesion	reference
*CAST*	Calpastatin	Yes	RA	5q15	rs27433; rs31250; rs754615; rs9667	Focal adhesion	[18], [57]
*ITGA4*	VLA-4, integrin alpha 4	-	-	2q31.3	rs12690517; rs155095; rs3770138; rs4667319	Adhesion molecule	[58], [59]
*ITGB1*	VLA-4, integrin beta 1	-	-	10p11.2	rs11009157; rs2153875; rs3780871;	Adhesion molecule	[7]
*ITGB2*	LFA 1, integrin beta 2	-	LAD	21q22.3	rs11559271; rs235326; rs7283236	Adhesion molecule	[60]
*PECAM1*	Platelet/endothelial cell adhesion molecule (CD31)	-	CP	17q23	rs1131012; rs13306812; rs6808	Adhesion molecule	[19], [61]
*PTEN*	phosphatase and tensin homolog	-	DM2, Cancer	10q23.3	rs10490920; rs2299939; rs2673836; rs532678	Negative regulator	[62]
*PTPN11*	protein tyrosine phosphatase, non-receptor type 11	Yes	-	12q24	rs11066320; rs11066323; rs7977332	Negative regulator	[63], [64]
*PTPRC*	protein tyrosine phosphatase, receptor type, C	-	MS, AIH	1q31-q32	rs10800584; rs1326269; rs17612648; rs1998843	Focal adhesion	[13]–[15], [65]
*PXN*	Paxillin	Yes	-	12q24.31	rs1634815; rs3742039; rs4767884	Focal adhesion	[12]
*SELE*	selectin E	-	SLE, RA	1q22-q25	rs5361	Adhesion molecule	[20]
*SELP*	selectin P (CD62P)	-	CP	1q22-q25	rs3917647; rs6131; rs6136	Adhesion molecule	[21], [22]
*SRC*	v-src sarcoma viral oncogene homolog	-	-	20q12-q13	rs6018199; rs6018257	Focal adhesion	[66]
*TYK2*	tyrosine kinase 2	-	SLE	19p13.2	rs12720214; rs2304256; rs280519	Activation of integrins	[11]
*VCAM1*	vascular endothelial adhesion molecule-1 (CD106)	-	Joint destruction	1p32-p31	rs3176878; rs3181088; rs3176860	Adhesion molecule	[67]

* Linkage regions according to Osorio et al. [5].

LAD = leukocyte adhesion defect; SLE = systemic lupus erythematodes; DM = diabetes mellitus; AIH = autoimmune hepatitis; CP = cardiopathy.

Our aim was to investigate genetic variants in these 14 genes for association with RA. We applied a multi-step approach, including two consecutively genotyped French RA family trio sets for discovery and replication. An additional set of 207 European RA family trios was analyzed to assess broader applicability of detected association effects in different populations. Overall, N_families_ = 407 family trios were genotyped. Possible functional aspects of identified associated SNPs were assessed by analysis of differential allelic expression (DAE) and respective eQTLs (expression quantitative trait loci). Losses and gains of possible transcription factor binding sites (TFBS) due to presence of SNPs was analyzed *in silico* and functional *in vitro* studies publicly available from the ENCODE project were reviewed [24].

## Materials and Methods

### Gene and marker selection

We selected genes in cellular adhesion processes following a thorough analysis of literature and databases at the time of candidate selection. For this purpose we used MeSH (medical subject headings) search terms in the PubMed database, available information on biological pathways (e.g. KEGG pathways, http://www.genome.jp/kegg/), gene ontology terms (http://www.geneontology.org/), and genetic databases such as OMIM (Online Mendelian Inheritance in Man, http://www.omim.org/), GDPinfo (genomics and disease prevention information system, currently accessible at http://hugenavigator.net/HuGENavigator/home.do), and GAD (genetic association database, http://geneticassociationdb.nih.gov/). Genes associated with autoimmunity and genes located in genomic regions linked to RA in genome-wide linkage analysis were preferred [10]. We note that our selection of genes must be considered essentially subjective. It represents a subset of genes for each of which a plausible link to cellular adhesion processes and to immune diseases is given.

Haplotype structure of each gene was analyzed by Haploview [25] and SNPs located in different LD (linkage disequilibrium) blocks were selected. SNPs with previously published disease associations or known functional consequences (e.g. non-synonymous coding SNPs) if existent were preferred. Information on SNPs was retrieved from databases EnsMart (http://www.ensembl.org/), PupaSNP [26], UCSC (http://genome.ucsc.edu), HapMap (www.hapmap.org), and dbSNP (http://www.ncbi.nlm.nih.gov/SNP/).

### Probands

Three sets of family-trios, each trio comprising an RA patient ( = affected individual) and both parents, were genotyped. Detailed characteristics of the first (French discovery set), second (French replication set), and third (multinational European replicaton set) cohort are described elsewhere [27], [28]. Briefly, the French discovery and French replication set each consisted of 100 family trios of French Caucasian origin with mostly similar characteristics. The third set consisted of 207 additional European Caucasian families, originating from France, Italy, Portugal, Spain, the Netherlands, and Belgium. Table 2 displays clinical information on all three cohorts as published previously [28]. All affected individuals fulfilled the American College of Rheumatology 1987 revised criteria for RA [29].

**Table 2:** Characteristics of rheumatoid arthritis (RA) index cases from the investigated samples.

	discovery set (N = 100)	replication set (N = 100)	multinational replication set 3 (N = 207)
**Population**	French Caucasian	French Caucasian	European Caucasian
**Females (%)**	87	90	86
**Mean age of disease onset (y)**	32 (±10)	31 (±6)	30 (±9)
**Mean disease duration (y)**	18 (±7)	16 (±8)	8 (±7)
**patients with bone erosions (%)**	90	79	70
**RF^+^ patients (%)**	81	76	73
**Patients carrying >1 HLA-DRB1 shared epitope allele (%)**	78	80	Not available

N = number of families; RF^+^ = rheumatoid factor positive.

### Ethics Statement

All individuals provided written informed consent and the study was approved by the Ethics Committee of Hôpital Kremlin-Bicêtre (Paris, France) according to the principles expressed in the Declaration of Helsinki.

### Analysis strategy and power calculation

In our multi-step approach all SNPs were genotyped in the first sample set, the French discovery set. Markers with a significant association with RA (uncorrected p value<0.05) were then genotyped in the second sample set of the same homogeneous population origin, the French replication set. As the replication set was highly similar to the discovery set regarding ethnicity, phenotyping procedure, and clinical characteristics, association in the replication set can be considered a successful replication of associations. When evidence increased in favor of an association, i.e. the p-value of the association decreased in the combined analysis of both sets, markers were genotyped in the third sample set, the multinational European replication set. As the third set is more heterogeneous due to its multiethnic origin, additional association in set three can be considered informative for the general relevance of an identified association.

For a marker with 35% minor allele frequency, we had 80% power to identify a genotype relative risk of 1.66, 1.44, and 1.29 within 100, 200, and 407 trio families in the TDT test, respectively. For a marker with 10% minor allele frequency, we had the same power to identify a genotype relative risk of 2.06, 1.7, and 1.47 within 100, 200, and 407 trio families in the TDT test, respectively. Power calculation was done using the R add-on package “powerpkg” using formula as described [30].

### Extraction of DNA and RNA

Genomic DNA and/or RNA were purified from fresh peripheral blood leukocytes or from Epstein-Barr virus (EBV) transformed cell lines for differential allelic expression analysis using standard methods.

### Genotyping

Genotyping was carried out using the Genosnip technique by Bruker Daltonics [31]. PCR primers were designed using MuPlex Vs 2.2 [32]. Primer design for SBE (single base extension) was carried out using PrimExtend, an in-house software tool based on CalcDalton [33]. Primer-sequences are shown in Table S1. Samples of the third set were genotyped by TaqMan 5′ allelic discrimination assays (Applied Biosystems, Foster City, CA, USA) following manufacturer's protocols. In quality control Mendel's 1^st^ law of inheritance had to be fulfilled in at least 95% of all children. Hardy-Weinberg Equilibrium (HWE) in non-transmitted controls had not to be violated in a chi-square test with 1 degree of freedom (p>0.01), all SNPs fulfilled these criteria. Non-transmitted controls refer to a genotype comprising of the two alleles that were not transmitted from the parents to the child. Individual-wise genotype call-rate in any set of family trios was always at least 94%.

### Association analysis

Linkage and association analyses were performed using the Transmission Disequilibrium Test (TDT) [34]. The TDT compares the transmission of SNP alleles from heterozygous parents to affected offspring with a transmission ratio of 50% as expected by Mendel's 1^st^ law. The allelic odds ratio (OR) compares differences in genotype distribution between RA cases and ‘virtual controls’ reconstructed from non-transmitted parental alleles. For gene-wide haplotype analysis the software Haploview 4.1 was used [25].

### Differential allelic expression (DAE) analysis

Complementary genomic DNA (gDNA) was derived from EBV immortalized B cells of individual probands (N = 52) and screened for individuals heterozygous for rs6136 applying Genosnip (see genotyping for details and Table S1 for primers). Total RNA of samples (N = 7) was extracted and reverse transcribed using standard protocols. Three of these samples (tagged with prefix “RA”) were derived from RA patients, which was not the case for samples termed 1–4. cDNA was genotyped applying Genosnip technology similar to gDNA with PCR primers designed to span exons (for-CTTCAGGACAATGGACAGCAGTA, rev-TCTTAGCAAAGCCAGGAGCG). Such primers make efficient co-amplification of gDNA unlikely. Correct PCR product size was controlled by agarose gel electrophoresis. For each cell line, three replicates were analyzed. Each single replicate was quantified at least in triplicates applying Genosnip and averaged. As the Genosnip method results in quantitative signals for the genotyped alleles, data from genotyping heterozygous cDNA can be used to quantify gene expression in an allele specific manner. To identify DAE, the allelic ratio of genotyped cDNA is compared with the allelic ratio found in gDNA, which is expected to be around one. For robust quantification, signal to noise ratios were used and the natural logarithm of the ratios was calculated. We applied ANOVA to globally identify if allelic ratios differed in cDNA from cell lines and gDNA (implemented in the statistical software R (http://www.R-project.org) applying the R add-on package “car”). Then, we used Dunnett's test to identify “regulated cell lines”, i.e. cell lines where DAE is present. This was defined to be the case when the allelic ratios of cDNA was statistically significant different from the allelic ratios of gDNA according to Dunnett's test (applying the R add-on package “multcomp” [35]). In the context of this manuscript, “cis-regulation” refers to DAE where regulated samples showed increased expression of always the same allele [36]. This might result from some direct mechanism which is not influenced much by other aspects of the state of a cell.

For verification of DAE by an independent method, purified PCR products (PCR-Purifying-Kit, Seqlab, Göttingen, Germany) originating from cDNA and genomic DNA of selected samples were analyzed by eurofins-MWG/operon (Ebersberg, Germany) with fluorescence-based sequencing (cycle sequencing technology with dideoxy chain termination using an Applied Biosystems ABI 3730XL-sequencer). In this analysis, relative peak height in sequencing traces was analyzed for evidence of DAE in an analogous fashion.

### eQTLs and gene expression analysis

Evidence for eQTL from public databases may be considered corroborative evidence for DAE. Therefore, eQTLs were assessed by comparing microarray expression data and genotyping data available via Genevar database (Version 3.5.0). Specifically, we used data from the pilot phase of The MuTHER Study [37] measured in lymphoid, skin, and adipose tissues derived from healthy female twin pairs.

Supplemental microarray gene expression data was accessed through the Gene Expression Atlas 2.0.7.5 05/11 (http://www-test.ebi.ac.uk/gxa/).

### Evaluation of allele effects on transcription factor binding sites

Genomatix SNPinspector software suite (v2.1, matrix library 8.3) was used to evaluate potential effects of SNP alleles on loss or gain of potential transcription factor binding sites (TFBS). Two scores, core similarity and matrix similarity, were analyzed. These scores are based on similarities between the highest conserved nucleotides of a predicted TFBS or a TFBS family matrix. Putative gains or losses of TFBS were deemed relevant for scores >0.8 [38].

Additionally, we reviewed *in vitro* data on TFBS at the genomic location of rs6136. Data are publicly available from the ENCODE project and accessible via the UCSC genome browser [24].

## Results

### Association study

Of 14 selected genes comprising 43 SNPs, two SNPs in genes *SELP* and *CAST* showed nominally significant association with RA in the discovery set of French RA family trios (Table S2). We also analyzed the association of gene-wide haplotypes in that sample set, but did not find significant haplotype associations after Bonferroni correction for the number of haplotypes found within the gene. We then genotyped SNPs rs754615 (*CAST*, p_TDT_ = 0.017) and rs6136 (*SELP*, p_TDT_ = 0.042) in the replication set of French RA family trios. *CAST* SNP rs754615 did not achieve significance in the replication set alone (Table 3). In contrast, SNP rs6136 was successfully replicated (p_TDT_ = 0.015, Table 4). As the direction of the association was the same in the discovery and replication sets, association of SNP rs6136 with RA was even stronger (p_TDT_ = 0.002) when analyzed in joined data from both sets. In consequence, SNP rs6136 qualified for genotyping in the European RA family trio set for evaluation of broader relevance. Here again rs6136 showed over-transmission of the major T-allele, but significance was only reached if analyzed in combination with the other two sets (pTDT = 0.003, Table 4). The power to detect the association of rs6136 with RA as reported in Table 4 for all sets combined was >95%. In subgroup analysis, the observed association for rs6136 was stronger in females (OR 0.58; 95%CI 0.4–0.8) than in males (OR 0.9; 95%CI 0.4–2.2) and stronger in patients with radiographic erosion (OR 0.71; 95%CI 0.4–1.4) than in those without erosion (OR 0.9; 95%CI 0.4–2.2). However, differences in odds ratios between subgroups were not statistically significant (p>0.3).

**Table 3:** Results of genetic association analysis for rs754615 – *CAST* (minor allele C).

	discovery set	replication set	joined discovery & replication set
**# informative parents**	98/200	87/200	185/400
**minor allele transmission**	61∶37	42∶41	103∶78
**Estimated Genotyping Accuracy (Mendel Errors)**	**100% (0)**	**100% (0)**	**100% (0)**
**TDT p-value**	**0.017**	0.94	0.07
**Minor allele frequency**	34%	40%	37%
**allelic OR (CI)**	1.7 (1.1–2.5)	1.02 (0.7–1.6)	1.34 (1–1.8)

Shown are results in all samples in a given sample set; Minor allele transmissions (transmitted alleles/untransmitted alleles) are shown. Informative parents: Number of parental individuals carrying a heterozygous genotype TDT: Transmission disequilibrium test considering informative parents, only. OR (CI) allelic odds ratio and 95% confidence interval of the minor allele when considering all parents (transmitted vs. non-transmitted alleles). Minor allele frequency relates to controls (non-transmitted alleles).

**Table 4:** Results of genetic association analysis for rs6136 – *SELP* (minor allele G).

	discovery set	replication set	joined discovery & replication set	multinational replication set	all sets combined
**# informative parents**	34/200	32/200	66/400	80/412	146/812
**minor allele transmission**	11∶23	9∶23	20∶46	35∶45	55∶91
**Estimated Genotyping Accuracy (Mendel Errors)**	**100% (0)**	**99.1% (1)**	**99.6% (1)**	**100% (0)**	**99.8% (1)**
**TDT p-value**	**0.042**	**0.015**	**0.002**	0.26	**0.003**
**Minor allele frequency**	13%	12%	13%	12%	12%
**OR (CI)**	0.54 (0.3–1.1)	0.38 (0.2–0.8)	0.47 (0.3–0.8)	0.77 (0.5–1.2)	0.62 (0.4–0.9)

Shown are results in all samples in a given sample set; Minor allele transmissions (transmitted alleles/untransmitted alleles) are shown. Informative parents: Number of parental individuals carrying a heterozygous genotype TDT: Transmission disequilibrium test considering informative parents, only. OR (CI) allelic odds ratio and 95% confidence interval of the minor allele when considering all parents (transmitted vs. non-transmitted alleles). Minor allele frequency relates to controls (non-transmitted alleles).

### Differential allelic expression

**Figure 1:**
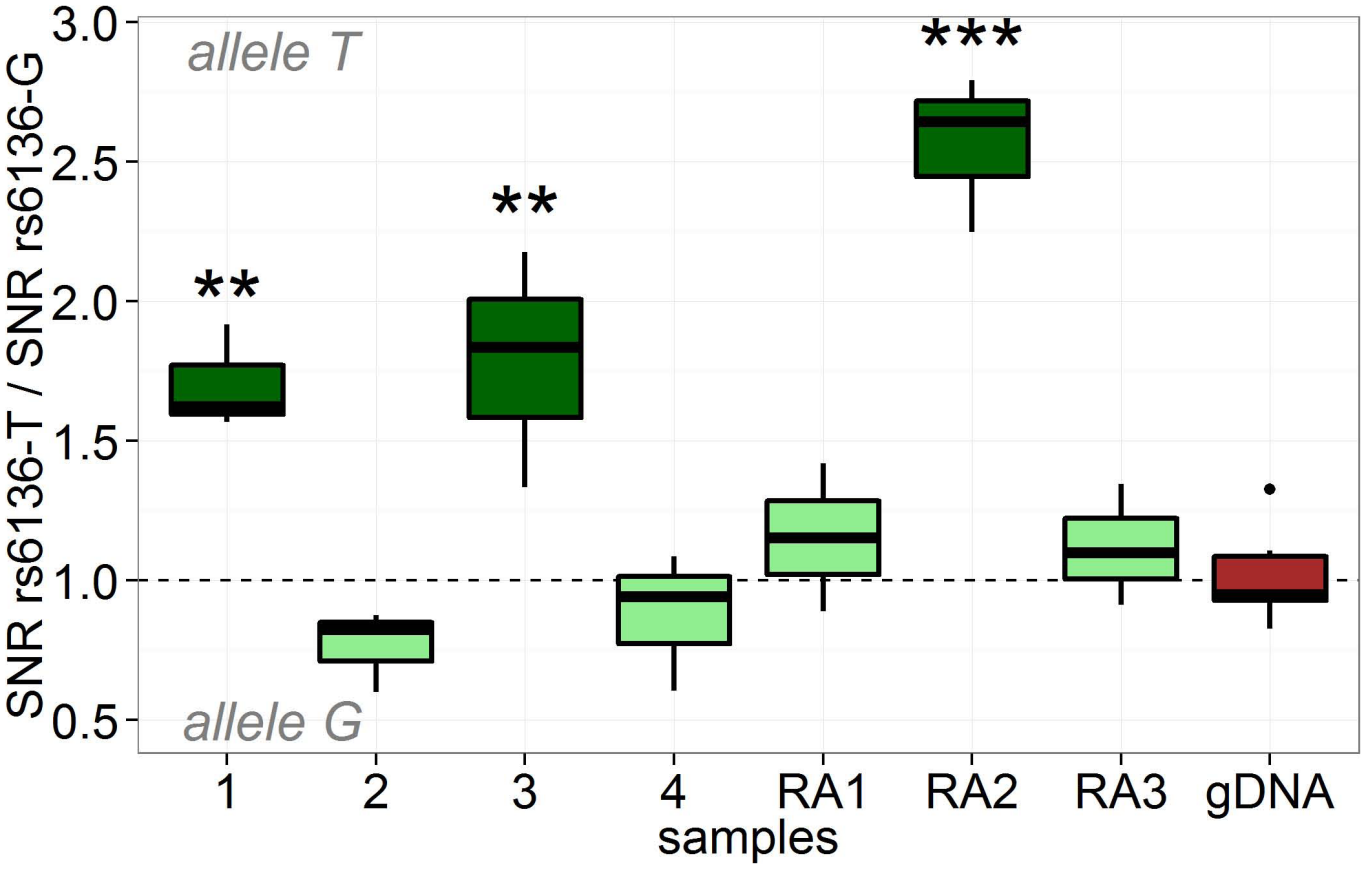
Cis-directed DAE pattern of rs6136 in the *SELP* gene. Green boxplots indicate samples resulting from allele specific expression analysis of cDNA. Samples tagged “RA” were derived from RA patients, which was not the case for samples 1–4; gDNA = allelic ratios of gDNA samples which served as reference. SNR = signal to noise ratio. Stars indicate cDNA samples with increased expression of transcripts carrying variant rs6136-T. ** = p-val<0.01, *** = p-value<0.001.

As *SELP* rs6136 was associated with RA in our multi-step RA family trio study we analyzed SNP rs6136 for differential allelic expression. We observed a cis-effect on expression levels of SELP for rs6136 (ANOVA p<10^−6^, figure 1). Dunnett's test revealed that three of the seven samples heterozygous for rs6136 individually showed significant DAE, always with the T-allele being higher expressed than the C-allele. The average fold change in regulated samples was 2. Direction of allelic expression fold changes was verified by Sanger sequencing (Table 5).

**Table 5:** DAE pattern of *SELP* SNPs for selected samples.

SNP	Sample	Allelic expression ratio genotyping	Allelic expression ratio sequencing	p-val
rs6136 (T vs. G)	1	1.78	1.36	0.0037
	RA2	2.51	6.18	<0.001
	RA3	1.12	1.65	0.97

Samples were measured with mass spectrometry-based genotyping and fluorescence-based sequencing; p-values refer to data from genotyping and are calculated by Dunnett's test analyzing significant differences between allelic fold changes between cDNA and gDNA.

**Figure 2:**
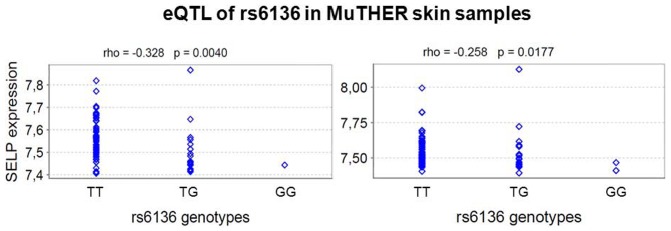
Cis-eQTL for rs6136 in the *SELP* gene in skin tissue from two sets of twins. Data were derived from The MuTHER Study [37] and analyzed by applying Genevar 3.5.0. These data successfully replicated DAE for rs6136. The two diagrams represent results from two skin sample groups derived from 76 and 84 female twins.

To further evaluate our finding of overexpression of the rs6136-T allele, we compared microarray expression data and genotype data available via the Genevar database. Within data from the pilot phase of The MuTHER Study [37] we observed a consistent cis-eQTL with overexpression of the rs6136-T allele (*SELP* probe: ILMN_1715417) in both (p_1_ = 0.004; p_2_ = 0.0177; figure 2). The rs6136 variant was the strongest cis-eQTL hit within available Genevar expression data in a region of at least 200 kbp up- and downstream of *SELP*. For the closest neighbouring gene *F5* (coagulating factor 5) no cis-eQTLs were detected.

The Genomatix SNPinspector software suite [38] was applied to calculate possible gains or losses of transcription factor binding sites (TFBS) for rs6136 and a SNP, rs9332575. SNP rs9332575 is in perfect LD (D′ = 1, r^2^ = 1) with exonic rs6136 within the Caucasian HapMap population, but located 40 kbp upstream of it (Table 6). The A-allele of rs9332575 corresponds to the T-allele of rs6136. Higher similarity values predict higher probability of transcription factor (TF) binding at this site. Table 6 shows several identified possible losses or gains of TFBS for rs6136 and rs9332575.

**Table 6:** Possible losses or gains of transcription factor binding sites for rs6136 and rs9332575.

SNP	alleles	loss/gain	TF family/gene	TF name	Core sim.	Matrix sim.
rs6136	T→G	loss	HNF1/HMBOX	Homeobox containing 1	0.77	**0.831**
rs6136	T→G	gain	SNAP/PSE	Proximal sequence element	**0.967**	0.756
rs6136	T→G	gain	E2FF/E2F	E2F transcription factor 1	0.785	**0.852**
rs9332575	A→G	loss	GATA	globin transcription factor 1	1	**0.938**
rs9332575	A→G	loss	HNF1	Hepatocyte nuclear factor 1 alpha	1	**0.841**
rs9332575	A→G	loss	EVI1	Ecotropic viral integration site 1 encoded factor	0.75	**0.819**
rs9332575	A→G	gain	ZFHX/AREB6	Atp 1a1 regulatory element binding factor 6	1	**0.984**

Prediction of altered transcription factor binding sites due to alleles of rs6136 and rs9332575. rs9332575 is a neighboring variant for rs6136 which is in perfect linkage disequilibrium. Core sim.: score for highest conserved nucleotides of a known TFBS. Matrix sim.: score for highest conserved nucleotides of a known TFBS family matrix. Similarity scores >0.8 indicate relevant predictions [38].

We also reviewed functional *in vitro* data on TF occupation near rs6136. Such data is publicly available via the ENCODE project [24]. For the genomic position of rs6136 we found an NFKB TFBS with the highest possible score in an assay based on chromatin immunoprecipitation (ChIP) followed by genome-wide sequencing in lymphocytes. Additionally, the locus appeared to be an open chromatin region as revealed through DNase I hypersensitivity assay, indicating that the locus would be accessible for TF.

## Discussion

Adhesion molecules are potential candidate genes for involvement in the complex immune disease Rheumatoid Arthritis. We selected 14 genes involved in cellular adhesion processes and investigated possible association with RA. Two of these genes, *CAST* (encoding calpastatin) and *SELP* (encoding P selectin), were associated with RA in our discovery set of French RA family trios. Association of SNP rs6136 in *SELP* with RA could be successfully independently replicated in our replication set of French family trios and was still significant when including a larger European Caucasian family trio cohort (p_Total_ = 0.003). Within all three sets, the major T-allele was over-transmitted to RA patients in TDT, which is robust against ethnic heterogeneity [39].

We compared our findings with data of a large meta-analysis of genome-wide association studies (GWAS) for RA [40]. Although rs6136 itself was not associated with RA (p = 0.86), several nominal associations (p = 0.023 to p = 0.043) were found within a range of +/−75 kb of the SNP. However, LD was rather low with those markers (R^2^<0.1). As association of rs6136 within our European Caucasian family trio cohort alone showed the same direction but did not reach significance, *SELP*-rs6136 might be of specific relevance in subgroups of RA patients with characteristics similar to our French discovery and replication cohorts. In particular, these two patient cohorts are known to be enriched for early onset RA and more severe phenotypes [41]. This should be considered when planning replication of the association of rs6136 in additional populations in addition to the power issues resulting from the relatively low minor allele frequency of rs6136 (Table 2).

We investigated SNP rs6136 for preferential expression of one of its alleles. Indeed, we observed preferential overexpression of the rs6136-T allele in three samples with an average fold change of 2.0 in regulated samples. Note, that there is a very high probability that DAE identified in at least three heterozygous individuals represents a true biological phenomenon [42]. However, not all samples heterozygous for rs6136 showed DAE, some appeared unregulated. This might indicate that in addition to the rs6136-T allele additional factors are necessary for upregulated SELP expression. Sampling effects might also offer an explanation. Alternatively, a SNP in non-perfect LD with rs6136 may be the true causative variant. However, we did not find any other polymorphism in or near the *SELP* gene which correlates with SELP expression more strongly than rs6136, even when accounting for common haplotype tagging SNPs. Interestingly, the highest differential expression was found in a cell line from an RA patient. However, overall, the pattern of DAE appeared to be rather similar between RA patients and non-RA patients (figure 1). Upregulation of SELP expression for carriers of rs6136-T was successfully replicated using data of The MuTHER Study [37] (figure 2). An association between rs6136 and SELP protein levels was also reported by the CHARGE consortium [43]. As this data is from different tissue and from different populations, this might hint at a general relevance of rs6136-T for increased gene expression of SELP.

Several TFBS were predicted to be changed either by variants of rs6136 or rs9332575, the latter a SNP in perfect linkage disequilibrium with rs6136. The highest scored (similarity score) loss of a TFBS was that of a GATA1 binding site for rs9332575 and the highest scored gain that of AREB6. While no RA relevant function is known for AREB6, GATA1 (globin transcription factor 1) plays a role in erythroid development and differentiation, but is also expressed in various cells, especially of hematopoietic lineage. GATA1 is reported to play a role in maturation of dendritic cells, an antigen presenting cell population able to activate naïve T cells during immune response [44].

We also found an NFKB TFBS by reviewing ENCODE *in vitro* data originally acquired by ChIP followed by genome-wide sequencing. An open chromatin structure at the site of rs6136 was found, indicating accessibility for TF binding. As NFKB is a TF predominantly activated during inflammatory processes and found to be especially relevant for RA pathology, NFKB might also be involved in increased selectin P expression levels in genetically predestined patients [45].

**Figure 3:**
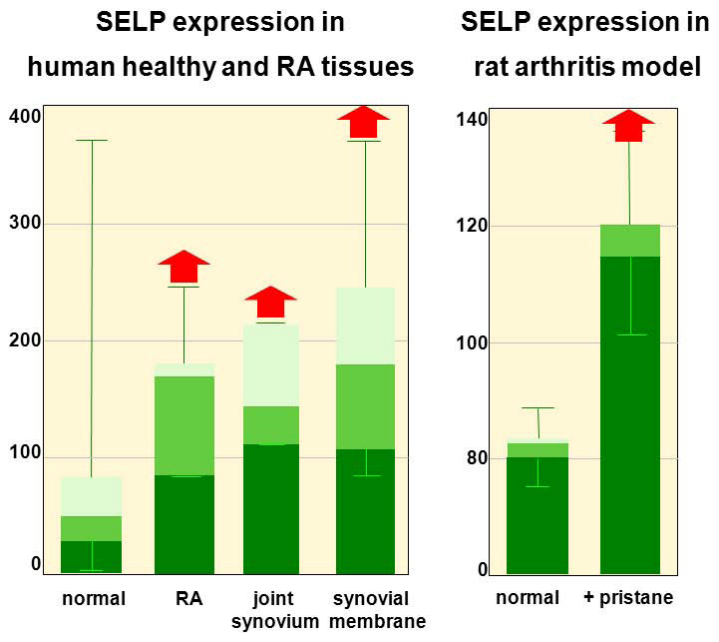
*SELP* expression profiles for human RA compared to healthy tissue and pristane induced arthritis in rat. Data derived from Gene Expression Atlas (E-GEOD-7307). N_normal_ = 679 (including healthy joint synovium and synovial membrane samples), N_RA_ = 5, N_joint synovium_ = 3 (all healthy), N_synovial membrane_ = 11 (N_normal_ = 6, N_RA_ = 5). N_rats-untreated_ = 5, N_rats+pristane_ = 5.

Within the Gene Expression Atlas (experiment E-GEOD-7307), *SELP* mRNA expression was found increased in two tissues most relevant for RA pathogenesis: joint synovium (p = 0.007, N = 3) and synovial membrane (p = 4×10^−10^, N = 11) (figure 3). *SELP* expression was also increased in RA patients (p = 0.033, N = 5) and in a rat model of pristane induced arthritis (experiment E-MEXP-782) (p = 0.047). Functional studies focusing on cis-regulation of *SELP* expression would be required to further corroborate our findings and possibly reveal the mechanism and consequences of *SELP* DAE.

The relevance of SELP in RA and other diseases is underlined by the finding of increased soluble SELP levels for many diseases, including atherosclerosis, in which soluble SELP is derived mostly from endothelial cells [44], and RA [46]. RA shows considerable comorbidity with cardiovascular diseases [23]. Soluble SELP correlates with RA disease activity [9], [47] and lower SELP levels are found during RA remission [48]. High levels of soluble SELP are predictive of future cardiovascular events [49]–[51]. Supporting this, Lee et al. reported SNP rs6136 being linked to high serum SELP levels within the Framingham Heart Study [52]. After adjustment for clinical factors such as hormone therapy, about 10% of altered SELP concentration in serum of participants is explained by the rs6136 variant. Individuals carrying the TT genotype display about double the SELP concentrations compared to individuals carrying the GG variant. These findings corroborate our own findings that rs6136 may be a functional SNP.

SELP is essential for recruitment, adhesion and migration of inflammatory cells into joint tissue and synovial membrane. Blocking of SELP expression with specific antibodies resulted in blocked adhesion [53], as well as reduced neutrophil recruitment [54]. In a *SELP* deficient mouse model of antigen-induced arthritis leukocyte-endothelial cell interaction was decreased [55]. Additionally, a chemical compound acting as SELP antagonist efficiently reduced inflammation in the rat adjuvant induced arthritis model [56].

The RA-associated major T-allele of rs6136 was linked to increased *SELP* expression. As a possible mechanism, we suggest that increased *SELP* expression might promote the increased recruitment of leukocytes to a site of inflamed tissue such as the synovial lining in RA. Subsequently, the inflammation may be prolonged or elevated, which might influence disease activity and severity. These processes related to *SELP* expression should be studied in more detail in the future.

In summary, we found *SELP* rs6136-T to be associated with RA and with increased expression of *SELP* mRNA. Genetically determined increased SELP expression levels might thus be a novel additional risk factor for RA.

## Supporting Information

Table S1 **PCR and SBE Primer for Genotyping of adhesion genes.** PCR primer designed with MuPlex 2.2, SBE primers designed with PrimeExtend (in house, request at peter.ahnert@gmx.net).
(DOC)

Table S2 **Results of Genotyping in French RA family trio set 1.**
(DOC)
