# Supplementary material for: Cellular Adhesion Gene SELP Is Associated with Rheumatoid Arthritis and Displays Differential Allelic Expression
Source: PLoS One. 2014 Aug 22;9(8):e103872. doi: 10.1371/journal.pone.0103872 (PMC4141704; doi:10.1371/journal.pone.0103872)
Supplement: Table S1 — PCR and SBE Primer for Genotyping of adhesion genes. PCR primer designed with MuPlex 2.2, SBE primers designed with PrimeExtend (in house, request at peter.ahnert@gmx.net). (DOC) [file pone.0103872.s001.doc]

**Table S1: PCR and SBE Primer for Genotyping of adhesion genes**.

| **Gene-SNP** | **PCR primer for.** | **PCR primer rev.** | **SBE primer** |
| --- | --- | --- | --- |
| CAST-rs27433 | ACGTTGGATGAAACCTTGAAACCTTTGACAC | ACGTTGGATGTCTTTAAACTTCTGGATTTTGG | bioCAGATACACATGGAAA(L)TAGTAATACTG |
| CAST-rs31250 | ACGTTGGATGACCAGTCTTTCAGCTTCTGT | ACGTTGGATGTACAGGCAGAATTTAAGGAGA | bioCATGAT(L)TAAGCCATGGGGA |
| CAST-rs754615 | ACGTTGGATGAGAGTACTTGTGTCTTTCCTCAG | ACGTTGGATGTCACTCACCAAGGTAGCC | bioCTCCTGTGTCGGA(L)GCTGTGT |
| CAST-rs9667 | ACGTTGGATGGTAAGTTTGCATTTGACTACATTG | ACGTTGGATGTAATCATATCCCTGTAGAATACAGTAG | bioCACCAGC(L)TAGGTTCTGGAGGA |
| ITGA4-rs12690517 | ACGTTGGATGACCACTTCAGCCAATTCTT | ACGTTGGATGTCCGACATATAGTTCACTTCTTC | bioATAATTTGAATAAAGGAAAAT(L)TTCCTAC |
| ITGA4-rs155095 | ACGTTGGATGCAAATCCTACCTCTGTCTTACTC | ACGTTGGATGCCTTGATATAACAAATCCCTTC | bioAAATGGCAATAAA(L)ATATCTCCCTC |
| ITGA4-rs3770138 | ACGTTGGATGAGACTCCTCCCCTCAAAA | ACGTTGGATGTCTCATGCCTAACGTCTCTC | bioCATATGGTA(L)ATTCATGCCATAAG |
| ITGA4-rs4667319 | ACGTTGGATGAAAGAGTGTGTGCATTAGAGC | ACGTTGGATGGTCTCACTTCTCTGTTGGAAA | bioGCAGACCTTGAAA(L)GCATAGTCC |
| ITGB1-rs11009157 | ACGTTGGATGAATGTAATGTACAGCCAAGCTA | ACGTTGGATGTTGTATCACCAGAGAGTGTAGG | bioAGCGCTG(L)CCAGCAACAT |
| ITGB1-rs2153875 | ACGTTGGATGATTGACCACAGTTGTTACGG | ACGTTGGATGTGATTGCAACTTTTAGTCTTGA | bioCTGATAATTTTTCTCACTTT(L)TTTTTGTT |
| ITGB1-rs3780871 | ACGTTGGATGATCCCTTCTTAAAGAGAAGAAAA | ACGTTGGATGTCTAGATGACCTTCTTTGATTTTA | bioTTTCAGTCAAT(L)TTTGCTAAGGTG |
| ITGB2-rs11559271 | ACGTTGGATGCAAGTTGTCCTCCAGGTG | ACGTTGGATGTCTTCTCAGGAGGAAATCG | bioCAGGAT(L)GCGCCCAGCTT |
| ITGB2-rs235326 | ACGTTGGATGGTAGAGTACAGGCTGGCATT | ACGTTGGATGATCCAGGAGCAGTCGTTT | bioGGCACTC(L)CACTGGGGAAG |
| ITGB2-rs7283236 | ACGTTGGATGAGATTTTCAGGAGTTTCAGGT | ACGTTGGATGTGAAAGGAAAACAACTATGAGTC | bioCCGCTCTCT(L)TCCCATCATC |
| PECAM1-rs1131012 | ACGTTGGATGAGCTAGCTACCTTCATTGACA | ACGTTGGATGGTCACTCACCCTAATTGTTATTT | bioTCAACTAGGTCACAA(L)GACGATGTC |
| PECAM1-rs13306812 | ACGTTGGATGAGGATCATTTGAGTTCTTGG | ACGTTGGATGTTTCTTATGATGCCCAGTTT | bioGGCAAAGTTC(L)ACTGATCGATTC |
| PECAM1-rs6808 | ACGTTGGATGATCTGTGCTTGTTCCACCT | ACGTTGGATGCAAATGGGAAAACCAGACTA | bioTGTGCG(L)TGCCTGAATGAAC |
| PTEN-rs10490920 | ACGTTGGATGACCGTGTGGTAATTTGAAGT | ACGTTGGATGGAGAATGCCCTAACAGCTT | bioAAATTATATTCACTC(L)GAAGTATACCTCC |
| PTEN-rs2299939 | ACGTTGGATGACGCCTGGCTAAATTGAT | ACGTTGGATGATTACAGCTTGGTTCACTTTTC | bioTGAGGGATATGA(L)TTGTTATGGAATG |
| PTEN-rs2673836 | ACGTTGGATGTGTGTTAGGGTTATTTTCGTT | ACGTTGGATGGTTAGCAATACTACAAACTCGTTC | bioGCAATATATTCTAATAAACC(L)AAAGTAAACTA |
| PTEN-rs532678 | ACGTTGGATGTCTGAAGAGTGGATAAGTCATTT | ACGTTGGATGATTTTGAAAGCATGAACACAG | bioAAGAAAACCAGTCC(L)AGTGTAAAGA |
| PTPN11-rs11066320 | ACGTTGGATGTAATTGGTGCACTAGGGAAG | ACGTTGGATGGCAGTAAGTTCTCAATCACCTC | bioGTGCTAAGTGCCAGCA(L)CTGACAG |
| PTPN11-rs11066323 | ACGTTGGATGCTCACTGAGTCCTTAAAATTGC | ACGTTGGATGAATTCCAGGCACTCACACT | bioCTTGGGTATCTC(L)AAACCTCAATTT |
| PTPN11-rs7977332 | ACGTTGGATGGTACAACTATGCCCAGTTAAGTTT | ACGTTGGATGTGAGTGAATGAATTTTGATAGG | bioCACTTTGGGAA(L)CTGAGGCA |
| PTPRC-rs10800584 | ACGTTGGATGAGCTCAATTGAAAATGGACA | ACGTTGGATGAATAGTTAGTAGTATCCGTATTCTTAAGTT | bioATTTAATGTGC(L)TATTAACCACCTGT |
| PTPRC-rs1326269 | ACGTTGGATGCAGACAATCAGCAAAATTATCA | ACGTTGGATGTTTTCCTTTCAGACACTTGAC | bioTAGTAGAGACCGAGC(L)TAAACAAATA |
| PTPRC-rs17612648 | ACGTTGGATGATTAACAGGATTGACTACAGCA | ACGTTGGATGATTGTCTGGACTAAGAGAAGTTGT | bioATTTTCTCTTT(L)AAAGGTGCTTGC |
| PTPRC-rs1998843 | ACGTTGGATGCCACCACTTCTGTGTAATTTT | ACGTTGGATGTTATTCAAATTGTTACCACCAC | bioCCTGCTGAGTACAC(L)TGCTTCC |
| PXN-rs1634815 | ACGTTGGATGCAATATATAAACAGAGAAAAGTAAGCAG | ACGTTGGATGGATCCATTCTTTCTTAGAGTCCT | bioGACGGC(L)ACAAACTCCCAA |
| PXN-rs3742039 | ACGTTGGATGCCTAGGGAGAGCTACAGGA | ACGTTGGATGCGACTGTGACCTAGAGACTTC | bioAAGCAGGG(L)GACCCCTCA |
| PXN-rs4767884 | ACGTTGGATGCCAGCTAAGTTCCCTCTGT | ACGTTGGATGAAACCACACATACCAGGAGA | bioTGGTGGATGA(L)TCGGGAGC |
| SELE-rs5361 | ACGTTGGATGAAGAGGCAAGAACCAGACTTA | ACGTTGGATGTCTAAAATCAAAGGCACTCAG | bioGCCTG(L)ACCAATACATCCTGC |
| SELP-rs3917647 | ACGTTGGATGGGCATTGCCTCATGTTAG | ACGTTGGATGGAGGTGGGAGAATTGCTT | bioAAAATATATATAGT(L)TCAGATGGGGATGA |
| SELP-rs6131 | ACGTTGGATGATAGGCAAAAGCAGTGAGC | ACGTTGGATGGCTTTTCTCCTTTCTCAGTATCT | bioAACACAG(L)CCATGGTTCCTTCA |
| SELP-rs6136 | ACGTTGGATGAAAGTGACTTCTTAACCCACA | ACGTTGGATGCAATCTGCTCTTTCCATTGT | bioTGGCAGGTT(L)GCACGGTAG |
| SRC-rs6018199 | ACGTTGGATGGGACCTTAGCTTCGTCTTAAA | ACGTTGGATGAAAAGACACAACCCCTGAC | bioTCACAAACACGA(L)GACCACTCC |
| SRC-rs6018257 | ACGTTGGATGTTTCCTCCCTCCTTCTGT | ACGTTGGATGTTGCCAAAATACCACCTG | bioACTGTGAGTG(L)GCAGAAGCC |
| TYK2-rs12720214 | ACGTTGGATGGACACAGCTGCATGTCCT | ACGTTGGATGCTGCAGGTGATCAGGAAG | bioCGGTCCTGTCTCC(L)TTCCCTC |
| TYK2-rs2304256 | ACGTTGGATGATGCTGACACAGTGCTCTT | ACGTTGGATGTGGGTGTTCAGGGTTCTA | bioGCCAAGGCTC(L)CAAGGCA |
| TYK2-rs280519 | ACGTTGGATGCCTGGGTGATCTCCTTCT | ACGTTGGATGGGGGATGACTGCTTCTCT | bioCCCC(L)ACCAGGAGGTACGA |
| VCAM1-rs3176878 | ACGTTGGATGCATTAATTGCATCCATTTTG | ACGTTGGATGAATGGCAGGTATTATTAAGGAG | bioGCACGAGAAGCT(L)AGGAGAAAAATA |
| VCAM1-rs3181088 | ACGTTGGATGGATGGCCAGTAGTATTTGCT | ACGTTGGATGGGGCCTAGATCTGTTGACTT | bioTCAGGCTGATGAAC(L)AAAGGAC |
| VCAM1-rs3176860 | ACGTTGGATGGGTTTCGTTTTATTGTACCG | ACGTTGGATGATGCACATACAACCAGCATA | bioAGCTT(L)AGCAACACATGCGG |

PCR primer designed with MuPlex 2.2, SBE primers designed with PrimeExtend (in house, request at peter.ahnert@gmx.net).
