# Supplementary material for: Cellular Adhesion Gene SELP Is Associated with Rheumatoid Arthritis and Displays Differential Allelic Expression
Source: PLoS One. 2014 Aug 22;9(8):e103872. doi: 10.1371/journal.pone.0103872 (PMC4141704; doi:10.1371/journal.pone.0103872)
Supplement: Table S2 — Results of Genotyping in French RA family trio set 1. (DOC) [file pone.0103872.s002.doc]

**Table S2: Results of Genotyping in French RA family trio set 1.**

| **Gene** | **SNP rs#** | **Minor allele** | **Major allele** | **Genotype rate** | **Cases HWE pval** | **Controls HWE pval** | **Minor allele frequ. cases** | **Minor allele frequ. controls** | **TDT pval** |
| --- | --- | --- | --- | --- | --- | --- | --- | --- | --- |
| CAST | 9667 | C | A | 96.3% | 0.345 | 0.815 | 17% | 19% | 0.589 |
|  | 27433 | A | C | 99.7% | 0.682 | 0.808 | 12% | 14% | 0.462 |
|  | 754615 | C | G | 100% | 0.982 | 0.777 | 46% | 34% | **0.017** |
|  | 31250 | C | G | 99.3% | 0.955 | 0.086 | 20% | 27% | 0.080 |
| ITGA4 | 4667319 | G | A | 100% | 0.539 | 0.554 | 30% | 37% | 0.105 |
|  | 155095 | T | C | 100% | 0.631 | 0.296 | 22% | 19% | 0.478 |
|  | 3770138 | T | C | 100% | 0.837 | 0.437 | 22% | 20% | 0.701 |
|  | 12690517 | A | G | 100% | 0.807 | 0.177 | 41% | 47% | 0.170 |
| ITGB1 | 2153875 | G | T | 99.3% | 0.624 | 0.493 | 24% | 27% | 0.493 |
|  | 11009157 | T | C | 98.7% | 0.547 | 0.118 | 24% | 27% | 0.666 |
|  | 3780871 | G | C | 93.7% | 0.584 | 0.574 | 12% | 17% | 0.275 |
| ITGB2 | 235326 | A | G | 99% | 0.253 | 0.749 | 28% | 28% | 0.929 |
|  | 11559271 | T | C | 100% | 0.990 | 0.352 | 27% | 24% | 0.503 |
|  | 7283236 | C | T | 99.7% | 0.005 | 0.378 | 42% | 40% | 0.849 |
| PECAM1 | 6808 | G | C | 97.7% | 0.768 | 0.667 | 45% | 49% | 0.399 |
|  | 13306812 | - | G | 98% | - | - | - | - | - |
| PTEN | 2299939a | A | C | 99.3% | 0.046 | 0.977 | 18% | 17% | 0.897 |
|  | 2299939br | A | G | 99.3% | 0.143 | 0.689 | 17% | 17% | 0.897 |
|  | 2299939cr | G | C | 99.3% | 0.046 | 0.577 | 18% | 18% | 0.898 |
|  | 2299939dr | T | G | 99.3% | 0.986 | 0.979 | 1% | 1% | 1 |
|  | 2673836 | C | T | 99% | 0.148 | 0.096 | 28% | 27% | 0.725 |
|  | 532678 | T | C | 98.7% | 0.206 | 0.052 | 33% | 31% | 0.565 |
|  | 10490920 | G | A | 99% | 0.192 | 0.165 | 12% | 12% | 0.882 |
| PTPN11 | 11066320 | T | C | 94.3% | 0.370 | 0.833 | 47% | 46% | 0.948 |
|  | 7977332 | G | A | 94.7% | 0.980 | 0.605 | 11% | 7% | 0.194 |
|  | 11066323 | T | C | 98.7% | 0.320 | 0.892 | 9% | 10% | 0.877 |
| PTPRC | 1326269 | A | T | 98.7% | 0.834 | 0.963 | 44% | 37% | 0.175 |
|  | 17612648 | - | G | 99% | - | - | - | - | - |
|  | 1998843 | T | C | 99.7% | 0.658 | 0.962 | 38% | 34% | 0.469 |
|  | 10800584 | A | G | 97% | 0.172 | 0.173 | 28% | 31% | 0.263 |
| PXN | 1634815 | C | G | 98% | 0.989 | 0.647 | 10% | 12% | 0.616 |
|  | 3742039 | C | T | 96.3% | 0.331 | 0.645 | 46% | 41% | 0.340 |
|  | 4767884 | A | G | 96% | 0.258 | 0.756 | 20% | 19% | 0.798 |
| SELE | 5361 | G | A | 94.3% | 0.803 | 0.860 | 29% | 23% | 0.251 |
| SELP | 6136 | G | T | 99.7% | 0.422 | 0.182 | 8% | 13% | **0.042** |
|  | 3917647 | G | A | 100% | 0.345 | 0.341 | 47% | 48% | 0.766 |
|  | 6131 | T | C | 100% | 0.401 | 0.413 | 18% | 18% | 0.896 |
| SRC | 6018199 | C | A | 95.3% | 0.985 | 0.444 | 14% | 15% | 0.675 |
|  | 6018257 | C | T | 98.3% | 0.157 | 0.760 | 12% | 9% | 0.336 |
| TYK2 | 280519 | C | T | 100% | 0.280 | 0.542 | 42% | 45% | 0.543 |
|  | 2304256 | T | G | 100% | 0.342 | 0.452 | 22% | 27% | 0.233 |
|  | 12720214 | C | G | 97.7% | 0.859 | 0.890 | 9% | 6% | 0.240 |
| VCAM1 | 3176878 | A | G | 94.3% | 0.512 | 0.425 | 16% | 15% | 1.000 |
|  | 3181088 | A | G | 94.3% | 0.721 | 0.892 | 13% | 19% | 0.149 |
|  | 3176860 | C | T | 97% | 0.051 | 0.218 | 38% | 34% | 0.399 |
